# Reports on the Separation of the Business of Teaching and Licensing

**Published:** 1847-07

**Authors:** 


### ECLECTIC DEPARTMENT,

### AND SPIRIT OF THE MEDICAL PERIODICAL PRESS.

*Reports on the separation of the business of Teaching and Licensing.* (*Reports adopted by the Medical National Convention; continued from page* 54.)

Two reports were submitted to the convention on this subject, both of which were accepted, and recommitted to a committee to report at the next meeting of the American Medical Association. Although these reports assume essentially the same ground, yet, as it would be invidious to select between them, each being excellent, we publish both. We are the more led to do this, inasmuch as the subject has excited considerable discussion, especially in this state, and we should not like to be suspected of withholding any communications from a desire to further partizan views.—Editor Buff. Jour.

*The Committee to whom was referred the following resolution, passed at the Medical Convention, held in the City of New-York, in May,* 1846—*viz.*

*Resolved,* That the union of the business of teaching and licensing in the same hands is wrong in principle, and liable to great abuse in practice. Instead of conferring the right to license on medical colleges, and state and county medical societies, it should be restricted to one board in each state, composed in fair proportion of representatives from its medical colleges and the profession at large, and the pay for whose services as examiners should in no degree depend on the n umber licensed by them—Respectfully report:

That they have carefully considered the resolution referred to them, and submit the result of their deliberation to the consideration of this Convention.

The resolution embraces two distinct subjects, each of which requires separate examination and discussion. *First,* it is stated that the union of the business of teaching and licensing is wrong in principle, and liable to great abuse in practice. There can be no question that the union of the business of teaching and licensing is liable to great abuse in practice, like the performance of all other important duties which devolve upon so fallible a being as man; but it is not quite so clear, or admits so readily of demonstration, that such union is wrong in principle. On the contrary, it would, at first sight, seem most natural to conclude, that those appointed to give instruction in the several branches, which constitute a suitable medical education, would be the best qualified to judge what proficiency the candidate for license had made, and whether he was qualified to practice or not. In this Convention it is believed that there will exist no difference of opinion, in relation to the abstract question, of the fitness of the teachers to judge of the qualifications of candidates for licenses of degrees. It will probably be admitted, that, taking teachers as a body, they are at least as well qualified to conduct the examinations, and to judge of the fitness or unfitness of candidates to be invested with the privileges of the profession, as any other class of practitioners. If this be conceded, it must also be conceded, that is not wrong in principle, or morally wrong, to unite teaching and licensing in the same hands. Then, if it be not wrong in principle, is it inexpedient to unite the two powers, because their union is liable to great abuse in practice? What are the abuses complained of, which, in the estimation of so many respectable members of the profession, call for a separation, to effect which, such sweeping changes are proposed to be made in the organization of our Medical Societies and Medical Colleges?

The abuse most loudly complained of, is, that in consequence of the great multiplication of medical schools in the United States, there exists such a competition for students, such a desire to have a long catalogue of names, and a goodly list of graduates, that almost every person who has gone through the prescribed course is allowed to pass, and that thus the people are imposed upon, and the character of the Profession degraded. It cannot be denied, that there is but too much truth in this charge, and too much ground for complaint. In perhaps every Medical School in the Union, the requirements for degrees are too low, and the honor, if such it may be called, is too unsparingly bestowed. But is it the fault of the colleges altogether that the requirements are so low? Has the profession thus far united in calling for higher attainments? The great complaint is that *some colleges* do not enforce rigidly and honestly the existing legal requirements, low as they are. There is reason to believe, that some of the oldest and most flourishing schools are as open to this charge as the obscure and comparatively unimportant ones. Hence much of the strong feeling now existing against all colleges in the profession. But while some Institutions enforce all the existing legal requirements in good faith, is it just to involve them in the same indiscriminate condemnation with those which abuse their privileges? The best remedy for the evil, the Committee are inclined to think, is to establish a more uniform and a more elevated standard of requirements in the several colleges for the doctorate, and to introduce into the board of examiners in such colleges, as do not already possess such a check, a sufficient number of gentlemen of the profession, not engaged in teaching, to insure that the requirements will be honestly enforced in every case.

For the present, at all events, it is deemed more prudent to reform the boards we have, than try the experiment of creating new ones —a reform is required, but not a revolution.

Another complaint is, that the pupils of teachers meet with more favor than others, and are allowed to pass, even when unqualified. There may be such cases; but this is a minor evil. It would be difficult to find a board of examiners, where favoritism would not, now and then, find access. Another ground of objection, rather insinuated than openly advanced, is, that the examiners may be influenced by the fees received for the degree or license, to pass persons they would otherwise reject. Most of the members of this committee have long been members of boards of examiners, and not feeling conscious of having, on any occasion, been influenced by such groveling considerations themselves, are, therefore, unwilling to believe, without the most positive proof, that such a charge, against professors, or censors, is well founded, or entitled to any consideration from this convention. If it be true, that the above-mentioned are the principal objections to the mode at present in use, to obtain licenses and degrees in medicine, (and they are almost the only ones the Committee have heard suggested, it would seem to be a matter of no great difficulty to correct any abuses that may exist, without having recourse to a new and untried tribunal, such as is proposed in the resolution referred to the Committee.

In favor of leaving the colleges in undisturbed possession of the privileges now possed by them, several arguments, entitled to consideration, may be adduced. 1st. Several of the institutions possess the privilege of conferring degrees, having the validity of a license to practice the different branches of medicine, as a vested right of which even the legislature cannot deprive them. Without their consent, therefore, no uniform rule applicable to all the States can be adopted, and uniformity is essential to the successful working of the reform contemplated.

2d. Under the present organization each school has it in its power to elevate or degrade the value of its diploma; and to make the possession of it an honor, or otherwise, according to the character of the school from which it emanates. The faculty of each college is interested in maintaining the reputation of the institution with which it is connected.

3d. The character of the graduates of an institution will, in a great measure, depend on the character, as men, and as teachers, of the Professors. When the Professors are men of elevated sentiments, the students insensibly imbibe the same high tone of feeling. The young graduate is proud of seeing the names of men whom he honors and perhaps venerates attached to his diploma; while teachers cannot fail to be gratified in attesting to the qualifications of diligent and well-deserving pupils. It may well admit of doubt whether it would be advantageous to break up this sympathy between students and their instructors.

4th. The teachers have the best opportunities of knowing the characters and capacities of candidates, from witnessing their general deportment in the class rooms, and at the several examinations to which, in most institutions, they are subjected, during the lecture terms. The necessity of good behavior, and regular attendance, for obtaining a degree, contributes greatly to secure that discipline and decorum in the class rooms so essential to the advancement of the pupil, and the success of the teacher. If the Professors were to be excluded from the board of examiners, or not to have a preponderating control, their influence over their classes would be greatly impaired. Every one knows that the conduct of a medical class depends almost entirely on that of the advanced students, and that among such of those as are not influenced by higher and more honorable considerations, the fear of the examinations operates as a powerful check on unruly behavior.

The Committee leavo it to the Convention to dedide, whether the arguments in favor of the present mode of granting diplomas in the colleges, are sufficient to counterbalance the abuses said to be connected with it.

The second division of the resolution proposes that "instead of conferring the right of license on medical colleges, and state and county medical societies, it shonld be restricted to one board in each State, composed in fair proportion of representatives from its medical colleges, and the profession at large, and the pay for whose services as examiners should in no degree depend on the number licensed by them."

Before entering upon any discussion of the merits of the suggestions contained in this part of the resolution, the Committee would bring to the recollection of the Convention, that at the present time there is scarcely a State in the Union, in which any license to practice medicine is required, and that consequently, if the degree of M. D., or the license of a Medical Society possess any value, the value is derived from the character of the source from which it emanates, rather than from any privilege it brings with it to the recipient. So far as the public is concerned, a simple certificate, signed by any association united for the purpose of teaching, if composed of men of eminence, would posess as much value and validity as a degree conferred by an incorporate college. The wish of the profession, seems to be to establish some uniform standard throughout the United States, so that the title of Licentiate in Medicine, or Doctor of Medicine, shall imply on the part of the possessor or recipient of such titles, the possession of some definite amount of knowledge and acquirement, both in his own profession and in general science. For the want of some such standard these medical titles are in some States respected, in others justly despised.

It is the opinion of this Committee, after a full consideration of the subject in all its bearings, that it is not at present expedient to recommend that the granting of licenses and degrees should be restricted to one board in each State. Where there already exist State and County Medical Societies, it is believed that it will be better and safer to improve the present organization and working of these, than to attempt a new organization.

The Committee cannot conceive of any plan by which a more full, and a more fair expression of the views of the profession can be obtained, than by the organization of County Societies and State Societies, to be composed of delegates representing said counties. There can scarcely exist a doubt that, if proper exertions were made by the united profession, a law might be enacted in every State of the Union for the incorporation of such societies, and requiring that a license should be obtained by every person before he could be recognized as a regular practitioner of medicine. But even if an act of incorporation could not be procured, voluntary associations upon the same general plan, would accomplish substantially the same purposes. They could determine the conditions on which persons would be admitted into the respective associations, which would soon have the force with the profession of a legal enactment. Without some such laws or associations among the respectable members of the profession, empiricism must prevail, as it does now, to a fearful extent.

As the wants of all classes of the community ought to be provided for, the Committee are of opinion that the requirements for license should not be so high as to place it beyond the reach of persons in moderate circumstances; whilst those for the degree of Doctor in Medicine should be such as the possession of so high an academic honor was designed to imply.

It may be said that the lives of the poor and humble are as valuable as those of the rich, and that, therefore, they ought to have as good physicians and surgeons. That may be all true; but we must take the world as we find it. In every country the great mass of the population are attended by medical men of inferior acquirements. The skill of a practitioner, however, does not depend so much on general accomplishments as on his strictly medical education; and is often happens that the comparatively illiterate general practitioner, is a better and safer medical adviser than the graduated and finished scholar.

If the requirements for license be placed too high, in the existing state of society in this country, the license will be altogether dispensed with, and persons will engage in practice without it. The question is not, between the present plan of medical education and the best that can be suggested; but between that which is now common, and the best that is practicable, and can at the present time be enforced.

From the general tenor of those remarks the Convention will gather that the Committee are not in favor of making any radical changes in the existing modes of obtaining licenses and degrees. They are of opinion that the Convention will be more usefully employed in recommending such measures as will correct the abuses that are known to exist, and in advising such alterations in the course and duration of study to be pursued in future by students, as will tend to elevate the medical profession and to render it more useful and more respectable. It is deemed inexpedient, at the present session, to introduce any subject, likely to mar the harmony of the Convention, or to lessen the influence of its deliberations.

In accordance with these views the Committee offer the following resolutions.

*Resolved*, That inasmuch as an opinion prevails to a considerable extent in the profession, that certain abuses have crept into some of the medical colleges, namely, that they confer degrees upon persons who have not fully complied with their own requirements, or on those who do not possess the requsite amount of knowledge to entitle them to such distinction, it is deemed expedient, by this Convention, in order to satisfy the just wishes of the profession, and to remove just grounds of complaint, that such colleges as do not already possess mixed boards of examiners, should consent to have associated with them in the examination for degrees, some members of the profession not engaged in teaching, or otherwise interested in such institutions.

*Resolved*, That the number of boards for granting licenses in the several States should be as limited as would comport with the convenience of examiners and candidates in each State.

*Resolved*, That it would conduce to the elevation and usefulness of the profession, that the regular and respectable members of it should form themselves into county or district societies, and that those societies should be represented in a State Medical Society, in such ratio to the whole number of each society as may be agreed upon as most proper—that each State Society should appoint such a number of boards for examining and licensing candidates as may be thought advisable, according to the size and population of the State.

*Resolved*, That when a college or colleges of medicine exist in a State, it be recommended to such colleges to invite a delegation from the State Society to be present at the time of the examinations for the admission of candidates for degrees, not for the purpose of embarrassing the faculty or candidates, but to satisfy the wishes of some portions of the profession, and relieve the institutions themselves from the imputations to which some of them seem to be at the present time exposed.

JAS. M'NAUGHTON, Chairman.
THOMAS COCK.

*The undersigned, members of the committee to whom was referred the following resolution of Dr. Bartles, viz:—*

(*Second Report on the same subject*).

*Resolved*, That the union of the business of teaching and licensing in the same hands is wrong in principle and liable to great abuse in practice. Instead of conferring the right to license on Medical Colleges and County Medical Societies, it should be restricted to one board in each State, composed in fair proportions of representatives from medical colleges and the profession at large, and the pay for whose services as examiners should, in no degree, depend on the number licensed by them,—respectfully Report

That the resolution referred to them, asserts a principle, and proposes a plan of action in conformity therewith—they will, therefore, consider it under these two aspects—and, first, as it relates to the principle involved in the union of teaching and licensing in the same hands.

If the resolution means to affirm that there is a moral wrong involved in this union, they cannot accord with it; they believe that medical teachers may confer the right to practice in good faith, and with a full conviction of the momentous responsibility which they assume, and they doubt not that there are medical faculties who perform this duty with strict integrity and impartiality, according to the received standards now recognized by the profession.

Nor can it be denied that, at the period when medical colleges were first organized in our country, the right of granting the license to practice, formed a wise and salutary feature in their policy. The best talent of the country was concentrated in the few medical schools which then existed. The number of students was comparatively small, and physicians were thinly scattered over the country.

Perhaps it would have been difficult at this juncture, to have vested the power of granting the degree, in any boards as competent to exercise it, as were the medical faculties of the several schools. To them the profession, with one accord, entrusted it, and that it was worthily exercised, according to the recognized standards of those times, we presume no one will deny.

We cannot concur, therefore, in the broad principle laid down in the resolutions before us, though we fully indorse the sentiment, that the union referred to is liable to great abuse in practice.

Within the past few years, the business of medical teaching has partaken largely of that spirit of active competition which is so peculiarly fostered by republican institutions. With the vast additions to our territory, the amazing increase of population, wealth, and general education amongst the people, there has been a corresponding activity in all of those pursuits which furnish honorable and profitable employment to those who engage in them. Amongst the most attractive of these to the medical man, is the business of public instruction.

Enthusiasm in the pursuit and diffusion of knowledge, ambition to excel in one of the most honorable of human avocations, a desire for social position and pecuniary emolument, all animate the breast of an aspirant for a professional chair. These motives are in themselves fair and praisworthy, and when maintained in their proper relations, and swayed by high moral principles, they constitute powerful incentives to meritorious exertion. Competition in the business of medical instruction does not necessarily imply a compromise of these principles. Under proper restrictions it may indeed be beneficial in stimulating the energies of institutions which might otherwise become dormant and inefficient. It has already accomplished much in this way, and is destined to be further beneficial in developing new sources of knowledge, and in perfecting plans of instruction.

Were men always disinterested, and true to the high impulses and generous sentiments of our nature, the interests of our profession would be in no danger from the most active competition. If the prevailing desire of all parties engaged in medical teaching was to elevate the standard of medical education, if rivalry between colleges consisted in efforts to extend and amplify their courses of instruction, and to establish a high reputation for scientific acquirements for their respective institutions, regardless of the popular demands for short sessions and superficial courses—then, indeed, would competition become the very life of progress and reform. But it is too often the case, that men engaged in the same pursuit, where the spirit of rivalry is active, and constantly stimulated, in their zeal for the interests or reputation of a favorite institution, will overlook considerations of public utility.

In corporations this tendency to deviate from a high standard is particularly strong, for in such bodies the responsibility of public acts is divided, and the sense of individual accountability is thereby weakened. Without attributing, therefore, to our medical colleges any extraordinary deviation from the line of integrity, we think it will be admitted that the circumstances under which they are now placed give them a downward tendency, and has produced, in some of them at least, a relaxation in their rules, and a consequent depreciation of the value of the degree.

A conviction of this general decadence was, we conceive, one motive which prompted the call of the first National Medical Convention held in New-York, and the reference of the subject under consideration to your committee by a large majority of that body, evinced their interests in its discussion.

When it is remembered that the medical diploma, as granted by the Colleges of the United States, confers the right to practice, and is not, as in many of the institutions of Europe, a mere Academic honor, which renders its possessor eligible for future examinations, it is not surprising that there should be throughout the medical community a deep interest in the just and impartial exercise of so important a trust. Nor should it be considered an improper interference with the rights of individuals or corporations, if this national body should inquire into the source from which this high power emanates; and into the means best calculated to restrict its undue exercise, if it should be abused to the injury of the profession.

The first question which presents itself in this connection is, what legal restraints now exist to the granting of the degree? or, in other words, what is the policy or practice of legislative bodies in this respect? We presume there will be no difference of opinion in this Convention that legislatures have recently been very liberal in granting charters to Medical Colleges. Nor will it be denied, that, in dispensing legal powers to these corporations, but little attention is bestowed by the mass of legislators, upon their influence and tendency upon the interests of science or of the profession. They are voted to applicants very much as are charters for banks, rail-roads, or manufactories; and legislative bodies rely upon the judgment and common sense of the people either to sustain or reject them, leaving each institution to stand upon its own merits.

That this is the tendency of modern legislation is evident from the fact, that irregular practitioners have been able to obtain legal powers to teach their doctrines, with a facility corresponding with their political or private influence at the seat of government. Our science receives, in fact, no substantial support from government, and, in some instances of recent date, where partial protection had been long extended, it has been withdrawn. As an evidence of this fact we would refer to a law passed by the Legislature of Missouri at its last session, one section of which declares, "Every person, or co-partnership of persons in this State, who shall follow the practice of law for a livelihood, in whole or in part, is hereby declared to be a lawyer; and every person, or co-partnership of persons in this State, who shall follow the practice of medicine for a livelihood, in whole or in part, is hereby declared to be a physician."

These remarks are not intended as casting any censure upon the legislatures of our country. As the fountains of law, the conservators of social order, and the media through which the popular will is expressed, these bodies demand, and should receive the respect of the citizen.

Your committee only desire to state their views of the actual relation which subsists between legislatures and medical corporations, and especially to direct attention to the fact, that the bodies which distribute these high powers, do not presume to judge of the qualifications of men to exercise them, or to institute any rigorous investigation into the manner in which they may act under them.

If this view of the case be correct, it becomes a question how far this liberal policy is conducive to the best interests of medical science, and whether some check should not be placed upon the power of granting licenses to practice, by the profession itself.

If, in the institution of medical colleges or schools, the granting of the diploma was restricted to its original objects, viz., to attest the proficiency of the student in the several branches of knowledge in which he may have been instructed, without conferring upon him any special privileges, or investing him with legal rights ; then the facility with which charters can be obtained, and the consequent multiplication of schools, would be comparatively harmless.

But it is easy to perceive that, under the present system, very defective schools may live and flourish, and even outstrip the more competent. All colleges possessing alike the power of conferring the right to practice—the diploma of the inferior institution bestows the same privileges at the cost of less time and money than those of a higher grade ; and as nearly all of these institutions are acknowledged by each other, it follows that a large class of students who may not choose to avail themselves of such facilities of instruction as are absolutely essential to ground them in the principles of medical science, will spend all, or a portion of the short period devoted to their studies in that school which will give them the most easy access to the ranks of the profession. On the other hand, inferior schools, struggling for an existence against their more powerful rivals, will use every effort which ingenuity can suggest to induce an attendance upon their courses.

Thus it is that medical education may be divested of its exalted aims, and be pursued more for the advantage of private individuals or institutions, than for the promotion of science—and for the good of humanity.

From the views now presented, it will be perceived that the undersigned concur in the proposition that, abuses consequent upon the union of the business of teaching and licensing in the same hands, are now apparent, and that unless some decided change in the policy of legislative bodies should be effected, or some measures should be instituted by the profession itself, to guard the exercise of this power, their abuses will continue to exist.

The undersigned will now proceed to the second branch of the resolution of Dr. Bartles, viz : "Instead of conferring the right to license on medical colleges, and on State and County medical societies, it should be restricted to one board in each State, composed in fair proportions of representatives from medical colleges, and from the profession at large ; and the pay for whose services, as examiners, should in no degree depend on the number licensed by them."

The undersigned prefer to discuss the general question of a remedy for the evils which they have endeavored to point out, without being bound by the specific proposition contained in this part of the resolution.

How far the entire separation of teaching from licensing, would be effectual in checking the progress of the evils complained of, they are not prepared to determine. Admitting, however, that this would be an important measure, would it be practicable, in the existing state of things, to effect it?

The first inquiry which here presents itself is, could the medical colleges be deprived of a right with which they have been legally invested, without their consent? This question is readily answered —they cannot. Nor can this right be interfered with any further than those who possess it may be disposed to modify or surrender it.

But it may be asked, suppose this power shall be abused, to the injury of the medical profession, and to the degradation of medical science? must its unrestrained exercise be permitted without an effort on the part of those most interested to check the progress of the evil? We answer, certainly not. And this brings us to the consideration of the means adapted to this important purpose.

We have before adverted to the inefficiency of legislative aid in sustaining sound scientific institutions, and have endeavored to point out the fallacy of a dependence upon bodies constituted as are our legislative assemblies in matters relating to science.

But there is a power more potent than that exercised by legislatures, or by the corporations which they may create; *it is the influence of combined and harmonious action directed to a special object, by the great body of the medical profession.* This power, which is popularly termed public sentiment, is, in this country at least, the most potential agency which can be brought to bear upon the errors or vices of the age, in whatever form they may be presented. It is, in fact, the source of law, and, in a republic, constitutes the mighty lever by which the powers of government are moved, and its decrees enforced.

Now, we must bring this great lever to bear upon the errors and abuses which exist in the medical profession, and if well directed, and skilfully applied, there cannot be a doubt that it will prove more effectual in removing them than appeals to legislation, or any partial attempt to remedy special evils.

The first step in this work, is to bring the medical profession together as a united body—to organize into a great family or brotherhood, bent upon the elevation and advancement of our common calling—to do, in a word, what this Convention at its first organization, as its first act, resolved to do, viz., "to institute a National Medicine Association, for the promotion of their interests, for the maintenance of their honor and respectability, for the advancement of their knowledge, and the extension of their usefulness."

To another committee has been entrusted the duty of presenting to this body a plan of organization adapted to this purpose; this done, and the initial step has been taken in the great highway of progress. Let the physicians in States and portions of States throughout our widely extended territory, combine together on the basis which this national association may recommend, and let these various subordinate bodies move in harmony with each other, and with the great central body, of which they form a part, and it is impossible that the deliberations or decisions of the profession thus united, can fail to command respect and allegiance from its individual members, from medical corporations, private associations, and from the public.

It will not be necessary, under such organization, to make any attempt to wrest from medical colleges their high powers. We have but to urge in the spirit of kindness, but with firm resolve, such changes in the existing plans of medical education, or in the modes of examination, as appear to be most imperatively demanded for the protection of our common interests, and for the good of humanity—and the colleges which are dependent on the profession for favor and support, will respond to the appeal.

Already we have an earnest of the sentiments of some of the gentlemen attached to these institutions, which inspires the hope that they stand ready to yield to the decision of the medical body. There are amongst the professors of our medical colleges, men, whose lives have been devoted to the improvement of science, and the advancement of every measure calculated to elevate the medical character ; in whose minds selfishness or avarice cannot be brought into competition with public utility, or with the behests of humanity. If, on an examination of the subject which is now forcing itself upon our attention, they become convinced that a change from the existing order of things is demanded, they will not be deterred from aiding in its accomplishment by any personal considerations. How far this feeling may pervade the entire body of professional teachers, we cannot of course determine, but that they are the sentiments of some wielding an extensive influence, and occupying the highest positions, we feel assured.

If, then, the National Medical Association should embody, as we believe it will, a large portion of the respectability and intelligence of the profession throughout the United States, and should assume a permanent and well disciplined form, we feel assured that any changes in the existing order of things which it may propose to the medical colleges of the country will meet with the most respectful consideration.

The interests and reputation of the colleges are, in fact, closely identified with the great body of the profession—the schools live and flourish by the support which they derive from the medical practitioners throughout the country—the great mass of their students are from the offices of these gentlemen, and their advice and influence must generally determine a student in the choice of his school.

The country practitioners form the great bulk of the medical community—they are the medical public, and the popular will is expressed through them. Their voices are now heard in tones of remonstrance against what they consider the abuse of the high power of conferring the right to practice medicine, and they are coming up here to this great national gathering, to adopt measures for the protection of our common interests. If, as one of these measures, the National Medical Association should ask that the body of the profession should have a voice in the selection of judges of the fitness of candidates for the doctorate, we cannot doubt that this right will be yielded, at least by those institutions who desire to mantain their connection with the National Association.

There is certainly nothing unreasonable in this request. When it is considered that in our profession the members are mutually dependent on each other for character and support, the privilege of deciding who shall be of the fraternity, is especially important; and if medical colleges, owing to any mal-administration of their affairs, or from being organized in a defective manner, throw into our ranks men who are incompetent to exercise the high responsibilities thus conferred upon them, it is the duty of the medical community to adopt measures for their own protection, and to ask for a participation, at least, in the exercise of this responsible duty.

The precise mode in which the profession may exercise a supervision over the granting of the degree—whether they shall establish independent boards of examination, or shall be associated with those now in force, is a question which your committee are not now prepared to discuss.

The object in this brief report is merely to re-affirm the existence of great and growing abuses in our systems of medical education, and to assert the rights of the great body of the profession to adopt such regulations for the government of the medical institutions of the country as their assembled wisdom may dictate.

The undersigned are not favorable to any sudden changes in the existing order of things—the positions and responsibilities of the National Association must be defined—abuses must be first exposed and great principles must be discussed before we can expect to, decide upon definite plans of action.

The report on the organization of National Medical Association, now before the convention, contemplates the exercise of the powers to which we have alluded, and has wisely established a standing committee of its body to have charge of the subject of Medical education. To that committee, this subject might, we think, be appropriately referred, with a view to future action.

The undersigned would further remark, that the report of the Committee on the Requirements for a Degree, also before the Convention, would, if adopted, exercise an important influence upon this question. If the curriculum of study is increased, and the terms lengthened, a very salutary check will be placed upon the too hasty or incautious exercise of the power of conferring the degree. And those Colleges who come into this arrangement may be viewed by the profession in such a light as to take away all occasion for complaint on this head.

The necessity for immediate changes in this branch of the system is thereby obviated, and the Convention may, we think, safely leave the question for future consideration.

With these views, the undersigned would respectfully submit the following preamble and resolution for the action of the convention.

*Whereas*, A general sentiment prevails in the medical profession that the active competition existing amongst the Medical Colleges of the Union has a tendency to lower the standard of professional requirement, and to depreciate the value of the degree,

*And whereas*, The facility with which charters for medical corporations are obtained from our state governments, exposes the medical profession to the continuance and increase of these abuses, inasmuch as these corporations possess alike the power of granting the license to practice;

*Therefore resolved*, That, in the opinion of this Convention, some additional checks to the exercise of this right should be established by the great body of the medical profession.

ISAAC PARRISH,
JAMES R. MANLEY,
THOMAS COCK,
JOHN W. FRANCIS.

---

*Report upon Registration of Births, Marriages and Deaths.*

The Committee appointed by the National Medical Convention, held in May, 1846, to consider the expediency and (if expedient) the mode of recommending and urging upon the several State governments the adoption of measures for a Registration of the Births, Marriages and Deaths of their several populations, respectfully Report:—

That in their deliberations upon this vitally important subject, the *expediency* of such a recommendation has not for a moment admitted of a doubt. They believe the medical profession of the United States would unhesitatingly and unanimously approve the recommendation, and that such a step, by their representatives assembled in this Convention, would receive a universal sanction.

A uniform and systematic Registration of Births, Marriages and Deaths, appears to be a measure somewhat difficult of accomplishment in populations like ours, and must, necessarily, rely upon the energy and intelligence of comparatively few to carry its provisions into effect, yet it is of such primary importance to the best interests of the people, as to justify our urging its adoption upon the several State governments, with the confident belief that when its merits are once fully understood, all will unite in its support.

With regard to the second branch of the subject submitted to us, the *mode* of recommending the adoption by the several States, of measures for this purpose, there would seem but one course for the Convention to pursue, and that is, to address the State governments upon the subject. To this end, your Committee have prepared a document in the form of an address, which they propose, if accepted should be signed by the officers of the convention, printed, and properly transmitted.

As this whole subject is one which can, in its primary aspect, be fully appreciated by but few others than the members of the medical profession, it must depend, in a great measure, upon *their* efforts for its successful accomplishments, and where such exceedingly desirable and valuable results are attainable, your committee will not suffer themselves to entertain the thought that it will be permitted to fail, through negligence on the part of their professional brethren in the different sections of country.

Your Committee respectfully submit the following resolutions for adoption by the Convention:—

*Resolved*, 1st. That it is expedient for this Convention to recommend to, and urge upon the various State governments, the adoption of measures for procuring a Registration of the Births, Marriages and Deaths occurring in their several populations.

*Resolved*, 2d. That a Standing Committee be appointed by the Convention to take a general charge of the subject, and report annually to the Convention.

*Resolved*, 3d. That the State Medical Societies be requested to assume the duty of carrying out the objects embraced in the first resolution; and that in those States where no organized societies exist, the delegates therefrom in the present Convention, be charged with the duty for their respective States, and report to the Standing Committee.

*Resolved*, 4th. That in procuring the Registration, the forms and nomenclature adopted, should be, as nearly as possible, similar to those prepared for, and reported to, the Convention.

*Resolved*, 5th. That the paper hereto annexed, be adopted as the voice of the Convention, be printed, and signed by its officers, and transmitted under their direction to all the State governments of the Union.

All of which is respectfully submitted.

JOHN H. GRISCOM,

GOUVERNEUR EMERSON,

A. CLARK,

CHAS. A. LEE,

JAMES STEWART.

Philadelphia, May 5th, 1847.

---

The United States National Medical Convention, assembled in the City of Philadelphia in May, 1847, desirous of the promotion of the true and vital interests of the people of their common country, in all their varied locations, circumstances and conditions, do respectfully recommend to the governments of the several States of the Union, the adoption of measures for a GENERAL REGISTRA

TION OF THE BIRTHS, MARRIAGES, AND DEATHS, which may occur within their respecive borders.

No effort need here be expended in elucidation of the more ordinary purposes for which such a Registration should be universally adopted, such as proofs of lineage, rights of dower, and bequests of property. The importance of these cannot but be perceived on the least reflection.

But there are reasons more profound and far reaching, results more important to the welfare and glory of man, obtainable by this measure, which not only justify, but demand its early adoption and thorough consummation.

There are two facts to be noticed in this connection, which may not be denied :—

*First.* Upon the circumstances connected with the three important eras of existence, birth, marriage and death, are dependent, to a very great extent, the physical, moral and civil condition of the human family.

*Second.* A knowledge of these circumstances is necessary for a full comprehension of important means for the certain advancement of the population of States, in prosperity and civilization.

To the political economist and vital statist, the laws which regulate and control the lives and destinies of the people of the present, cannot be a subject of indifference ;—to the legislator and statesman, ignorance of them is a bar to the full appreciation of their responsibillity to the people of the future. The *philosophy of increase of population* is intimately connected with, and dependent upon the proposed measure, and can be properly learned only from its facts and deductions. In countries longer settled than ours, this science has come to be one of profound importance to those who are called to legislate for the future as well as for the present. For example: The population of England has increased, as the census prove—and the excess of births over deaths leaves beyond a doubt—in a geometrical progressiou for forty years, and a rate by which, if continued, it will double every forty-nine years. Whether the means of subsistence keep pace with that increase, or whether the density of population will, ere long, be too great for its area, are important questions to be decided by theij own statesmen.

An increase of population has, however, nothing in it irresistible or inexorable; *it consists in nothing but an increase of the births over the deaths*—and will be suspended if the births cease to maintain the same ratio to the population; and the births may always be reduced rapidly, by retarding the period and number of the marriages, without taking into consideration the increase by immigration. Circumstanced as this country is now, with its millions of unreclaimed acres, its exhaustless resources of subsistence and wealth in its mountains and valleys, in its mines, rivers and forests, it would be judicious to invite, even with the vast immigration to be expected, rather than discourage, an increase of a native population, by encouraging early marriages, provided that thereby immorality or misery in any form, will not advance with them.

But before we can make any recommendations on this subject, or before we can even intelligently discuss it, we must have a knowledge of the facts as they are. By commencing a Registration now, our successors will be furnished with the necessary material in time for any exigency that may arise.

Conclusive evidence is furnished to us of the value of a well-digested system of Registration for the improvement of the people in their moral and physical condition, and in the length of their lives. From the facts obtained thereby, are deducible the rules and inferences of health, and the sources of disease and premature mortality—many of which need but be known to be avoided. Coincident with improvements in the health and condition of individuals, are increase of years, and advancement in private and public morals, and in the strength and virtue of the State.

Among the first communities to establish a system of Registration of Births, Marriages and Deaths, was Geneva, where it was begun as early as 159, and has since been continued with great care. The registers are there viewed as pre-appointed evidences of civil rights, and it appears that human life has wonderfully improved since they were kept. The mean duration of life increased more than *five times* from 1550 to 1833; with the increase of population, and more prolonged duration of life, *happiness also increased;* though with advanced prosperity, marriages became fewer and later, and thus the number of births was reduced, a greater number of infants born were preserved, and the number of adults—with whom lies the true greatness of the State—became larger. Towards the close of the 17th century, the probable duration of life was not 20 years—at the close of the 18th century, it attained to 31 years—and now it has arrived to 45 years; while the real productive power of the population has increased in a much greater proportion than the increase in its actual number, and, *Geneva has arrived at a high state of civilization.*

These results, so glorious for individuals, for the community, and for humanity, are derived from the better knowledge and understanding of the science of life and health, the data for which are furnished by the statistics of the Registers.

The information obtained by the Natural History surveys which have been made of many of the States of the Union, is directly interesting only to a very small number;—while the facts and inferences deducible from a sanatary survey and registration, interest and benefit, directly, the great mass of the people, for all are interested in their personal condition. Thus are produced in them more expanded views of the worth of life, and the necessity for its perservation; a more thorough appreciation of the importance of purity in the principle sources of its continuance, air and food; more attention to the comforts of dwellings and clothing, more refined sensibilities, greater energy, and a better regulated state of public and private morals. These results *have obtained in Geneva.*

In Prussia these measures are attended to in a mode deserving the highest commendation. Every fact relating to the health, lives and condition of the population, is there collected with great care by a central officer at Berlin, and published for the benefit of the people. The most beneficent results have accrued from the admirably arranged statistical returns made for several years past in England. Of more than one large town, but of Liverpool especially, it was ascertained that the mortality was great, and the average age at death of the population low, whereas before, the inhabitants had boasted of their salubrity and longevity. The registration has, to them, truly proved the means of increase of health and years, after removing from their eyes the scales which blinded them to their own destruction.

In many of the European states besides those mentioned, facts in connection with this subject are registered, and collated, in the most scientific and systematic manner, and, to use the language of a distinguished American statist, "whatever we Americans may say to the contrary, the average longevity, in many places, where these measures have been in operation, appears greater than with us," Indeed we have no little reason to apprehend that unless something is done to arrest the progress and pressure of the causes of premature mortality in this country, we shall be in danger of possessing only a very young and immature population. The average age of death in many of our large cities, as far as returns enable it to be shown, is under 20 years, a fact which can only be due to the unfavorable physical circumstances of the people, and their ignorance of the true means of living and avoiding disease.

The registers of the ancient Romans, which were preserved with great care, and recorded the births, sexes, periods of puberty manhood, age at death, etc., kept by order of Domitius Ulpianus, prime minister of Alexander Severus, afford us the means of ascertaining the mean duration of life in Rome nearly 2000 years ago, and comparing this with the results of estimates made at the present day in places where similar records are kept, we are thus enabled to establish the gratifying fact of the great extension of the average period of human life in various cities and countries.

Of the results obtainable by the suggested measure, in connection with the census returns now regularly made in each of the United States, not the least important and desirable are *tables exhibiting the probabilities or expectation of life.*

By this simple and elegant method, the mean duration of life, uncertain as it appears to be, and as it *is*, with reference to individuals, can be determined with the greatest accuracy in nations, and in still smaller communities. This is important not merely in reference to the payments of life annuities, and the business of life insurance, whose great value is but just beginning to be felt in this country, but it is of inestimable interest, as determining to individuals their probabilities of living in their different classes, occupations, locations, and habits. "As it might be expected from the similarity of the human organization, that all clases of men would *cæteris paribus*, live, on an average, the same number of years, it becomes important to ascertain whether this be the case, and if it be not, to determine to what extent life is shortened in unfavorable circumstances. The Life Table answers this purpose, and is as indispensable in sanatary inquiries as the barometer or thermometer, and other instruments, in physical research· Upon applying it to any number of well-selected cases, the influence of any external cause, or combination of causes, can be analyzed; while without its aid, and extended observation and calculation, we are liable to be misled at every step by vague opinions, well-concocted stories, or interested statements, in estimating the relative duration of life; which can no more be accurately made out by conjecture, than the relative diameters of the sun, moon, and planets of our system."*

If these things are so, and of their truth there cannot remain the shadow of a doubt, it is plain that with this measure are entwined the highest earthly interests of humanity, and it belongs to the legislators of the New-World, the guardians and custodians of the interests and glory of the American Republic, to consider well ere they longer postpone the adoption of a measure so essential thereto. "A comparison of the duration of successive generations in England, France, Prussia, Austria, Russia, America, and other States, would throw much light on the physical condition of their respective populations, and suggest to scientific and benovolent individuals in every country, and to the governments, many ways of diminishing the sufferings, and meliorating the health and condition of the people; for the longer life of a nation denotes more than it does in an individual,—a happier life—a life more exempt from sickness and infirmity—a life of greater energy and industry—of greater experience and wisdom. By these comparisons, a noble national emulation might be excited, and rival nations would read of sickness diminished, deformity banished, life saved—of victories over death and the grave,—with as much enthusiasm as of victories over each other's armies in the field; and the triumph of one would not be the humiliation of the other, for in this contention none would lose territory, or honor, or blood, but all would gain strength." (*Idem*).

*Fifth Annual Report of the Registrar-General in England.

